# Photoinhibition and protection of photosystem I

**DOI:** 10.1093/pcp/pcaf079

**Published:** 2025-07-20

**Authors:** Kintake Sonoike

**Affiliations:** Faculty of Education and Integrated Arts and Sciences, Waseda University, 2-2 Wakamatsu-cho, Shinjuku-ku, Tokyo 162-8480, Japan

**Keywords:** chilling sensitivity, electron transport, fluctuating light, redox regulation, photoinhibition, photosystem I

## Abstract

Over 30 years ago, when we first reported the selective photoinhibition of photosystem I (PSI) in chilling-sensitive plants at chilling temperatures, the inhibition tended to be regarded as a specific phenomenon observed only under unusual conditions. We have since learned that PSI can be photoinhibited under many different conditions. The inhibition was observed in isolated thylakoid membranes under low light, or with some mutant plants under fluctuating light, or with plants illuminated by a series of saturating light pulses. Apparently, the sensitivity of PSI to photoinhibition is an intrinsic property of this photosystem. To understand the mechanism of PSI photoinhibition, which is now known to occur universally in nature, a comparison of different types of PSI photoinhibition should certainly be useful. In this review, similarities and differences in the mechanisms of photoinhibition between different types of PSI photoinhibition, as well as the protection mechanisms from the inhibition, are discussed.

## Introduction

The term “photoinhibition” was originally defined as a decrease in photosynthesis under excessive light (Kok 1956). It has long been used as a synonym for the photoinhibition of photosystem II (PSII) (e.g. Aro et al. 1993), since the selective photoinhibition of photosystem I (PSI) *in vivo* was not reported until 1994. It was then discovered that PSI in chilling-sensitive plants such as cucumber suffers severe and selective photoinhibition of PSI under moderate light at chilling temperatures with a minor effect on PSII (Sonoike and Terashima 1994, Terashima et al. 1994). Following these reports, it was demonstrated that simple illumination of thylakoid membrane suspension with weak continuous light at room temperature for <1 h resulted in severe and selective photoinhibition of PSI both for cucumber and spinach, i.e. irrespective of the chilling sensitivity of the plant materials (Sonoike 1996b). It was obvious that the sensitivity of PSI to photoinhibition was an intrinsic property of this photosystem. Chilling sensitivity should be dependent on the impairment of some protective mechanisms from photoinhibition rather than the photoinhibitory processes in PSI complexes themselves.

Even after this discovery, however, photoinhibition of PSI has tended to be considered a peculiar phenomenon brought about only under limited conditions. This situation changed in the 2010s. First, it was found that photoinhibition of PSI was readily induced in the mutants of *Arabidopsis thaliana* defective either in cyclic electron transport or in state transition under “fluctuating light” at normal temperatures (Tikkanen et al. 2010). Such photoinhibition of PSI under high/low light cycle was observed in other mutants (e.g. Allahverdiyeva et al. 2013) or even in wild-type (WT) plants, although to a much lesser extent (e.g. Kono et al. 2014). PSI photoinhibition can also be brought about by illuminating plants with a series of saturating light pulses (e.g. Sejima et al. 2014).

The conditions that were reported to induce PSI photoinhibition look different from each other; chilling stress, thylakoid isolation, fluctuating light, and a series of saturating light pulses (Table 1). While the mechanisms of the inhibition seem to be somewhat different, there are certainly some common and essential aspects in the PSI photoinhibition as well. All the PSI photoinhibitions are dependent on oxygen and electron transport from PSII. The differences, on the other hand, can be ascribed to the difference in photon flux densities during photoinhibitory treatments at least in part. The mechanism of PSI photoinhibition is also totally different from that of PSII photoinhibition. In that regard, the difference in the mechanisms should be attributed to the difference in the redox potential of the two photosystems. Most of the photosynthetic reactions are regarded as redox reactions, and so are some of the protective mechanisms against photoinhibition. Furthermore, some of the reactions producing reactive oxygen species (ROS), the damaging species in the process of PSI and PSII photoinhibitions, are also redox reactions. In this review, the emphasis is put on the similarities and differences in the mechanisms of photoinhibition among different types of PSI photoinhibitions, as well as between PSI and PSII photoinhibitions. Characteristics of the redox reactions that are related to the mechanism of photoinhibition, as well as the mechanisms protecting photosystems from photoinhibition, are the key to understanding photoinhibition. As for the PSI photoinhibition under specific conditions, please consult with the earlier reviews focused on each aspect; chilling-induced and thylakoid isolation-induced (Sonoike 2006, 2011), fluctuating light-induced (Yamori 2016), and saturating pulse-induced (Miyake 2020) PSI photoinhibitions.

**Table 1:** Typical conditions inducing photoinhibition of PSI and its characteristics

	Light period duration	Photon flux densities (μmol m^−2^ s^−1^)	Damaging oxygen species	Site of inhibition
Isolated thylakoid membranes	Continuous	80	Hydroxyl radical	FeS
Chilling stress in chilling-sensitive plants	Continuous	200	Hydroxyl radical	FeS
Fluctuating light	5 min/1 min	50/500	Hydroxyl radical?	FeS
Repetitive saturating light pulses	10 s/0.8 s	0/6000	Hydroxyl radical? Singlet oxygen?	Chlorophyll near reaction center?

## The Basic Mechanism of PSI Photoinhibition

**Figure 1:**
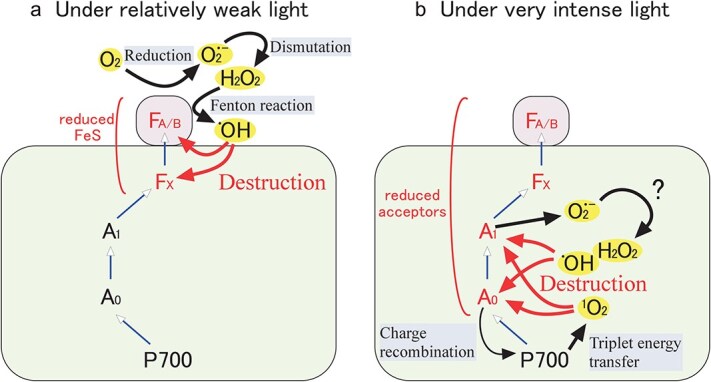
The basic mechanism of PSI photoinhibition. Under relatively weak light photoinhibitory treatment, i.e. with isolated thylakoid membranes, under chilling stress in sensitive plants, or under fluctuating light, electrons from PSII reduce FeS of PSI, which in turn reduce molecular oxygen producing O2˙^−^, H_2_O_2_, and finally ˙OH destructs FeS (a). Under very intense light, such as repetitive pulses of saturating light, earlier electron acceptors are also reduced and that may bring about the reduction of oxygen as well as the production of ^1^O_2_ through charge recombination (b).

Let’s start with the simplest condition to induce PSI photoinhibition, i.e. illumination of isolated thylakoid membranes without stromal components. If we expose suspension of thylakoid membranes isolated from spinach to continuous weak light at 80 μmol m^−2^ s^−1^ for 90 min at 25 °C, photochemically determined contents of P700, the reaction center chlorophylls of PSI, decrease to one-third of the original level (Sonoike 1996b). Considering the fact that the inhibition was totally suppressed in the absence of oxygen as well as by the addition of inhibitors of electron transport such as 3-(3,4-Dichlorophenyl)-1,1-dimethylurea (DCMU) or 2,5-dibromo-3-methyl-6-isopropyl-*p*-benzoquinone (DBMIB), it is reasonable to assume that oxygen reduction on PSI and subsequent production of ROS are the cause of photoinhibition. The necessity of electron flow from PSII was also shown by the pH dependency of PSI photoinhibition, which was well explained by that of the electron transport activity of PSII (Sonoike 1995). Here, it is important to note that the term ROS is rather ambiguous, and it must specify the actual damaging species that brought about the photoinhibition of PSI. The product of oxygen reduction is superoxide anion radical (O_2_˙^−^), which is then spontaneously or enzymatically dismutated to hydrogen peroxide (H_2_O_2_). In the presence of reduced metal ions, hydrogen peroxide is converted to hydroxyl radical (˙OH) by the so-called Fenton reaction, and this ˙OH is the most reactive and short-lived species among ROS (for a review of ROS in photosynthesis, see Asada 1999 and see also Fig. 1a).

Interestingly, the addition of methyl viologen (MV), an artificial electron acceptor of PSI that is known as a source of O_2_˙^−^ through its autoxidation activity, totally suppressed the PSI photoinhibition in isolated thylakoid membranes (Sonoike 1996b) as well as that induced by series of saturating light pulses (Takagi et al. 2016). Thus, O_2_˙^−^ itself is not a damaging species. Furthermore, the addition of H_2_O_2_ to thylakoid membranes in the dark does not cause any inhibition even at unphysiologically high concentrations such as 30 mM (Sonoike 1996b), suggesting that H_2_O_2_ is not the damaging species as well. From the fact that the addition of *n*-propyl gallate, a scavenger of ˙OH, partially protects PSI from photoinhibition (Sonoike 1996b), it is reasonable to assume that ˙OH is the actual damaging species in the process of PSI photoinhibition. Since ˙OH is produced by the Fenton reaction between H_2_O_2_ and reduced metal ions while H_2_O_2_ itself is not damaging, the key component in the process of inhibition should be the reduced metal ions. In fact, the addition of H_2_O_2_ under illumination totally abolishes the PSI activity, suggesting that reduced metal ions must be generated by the illumination. The PSI reaction center complexes contain three iron–sulfur clusters (FeS, namely, F_A_, F_B_, and F_X_) as electron acceptors, each containing four redox-active iron atoms. Thus, the mechanism of the damaging process is considered as follows (Fig. 1a). The first step is the reduction of the electron acceptors of PSI by the electron flow from PSII. In the absence of efficient electron flow to ferredoxin and NADPH pool (which is lacking in isolated thylakoid membranes), the reduced FeS donates electrons to oxygen, producing H_2_O_2_ through O_2_˙^−^. The Fenton reaction between H_2_O_2_ and reduced iron in the FeS produces ˙OH, which immediately destroys FeS. This general scheme of PSI photoinhibition in isolated thylakoid membranes can be a starting point to consider the mechanisms of PSI photoinhibition induced under different conditions.

## Comparison of the ROS Production in Different PSI Photoinhibitions

The requirement of oxygen for inhibition was also demonstrated in PSI photoinhibition *in vivo*. Chilling-induced photoinhibition of PSI was totally suppressed in the nitrogen atmosphere (Terashima et al. 1994). The requirement of oxygen was also reported in PSI photoinhibition induced by fluctuating light (Kono et al. 2014) or by a series of saturating light pulses (Sejima et al. 2014). The involvement of oxygen in the process of PSI photoinhibition is apparently universal among different conditions used to induce photoinhibition. Similarly, the inhibition of electron transport from PSII was also shown to protect PSI from photoinhibition *in vivo* as well as *in vitro*. For example, when the PSII activity of cucumber leaves was inhibited beforehand by the dark-chilling treatment (Kudoh and Sonoike 2002b) or UV treatment (Zhang et al. 2016), subsequent light-chilling treatment did not induce photoinhibition. The addition of DCMU also protects PSI from photoinhibition under fluctuating light conditions (Suorsa et al. 2012). The importance of the reduction of electron carriers of PSI has been repeatedly emphasized in all types of PSI photoinhibition. It is natural to assume that the electrons from PSII through cytochrome *b*_6_*f* are used for the reduction of molecular oxygen on PSI acceptor side to produce ROS. So far, the mechanisms of the PSI photoinhibitions seem to have much in common.

**Figure 2:**
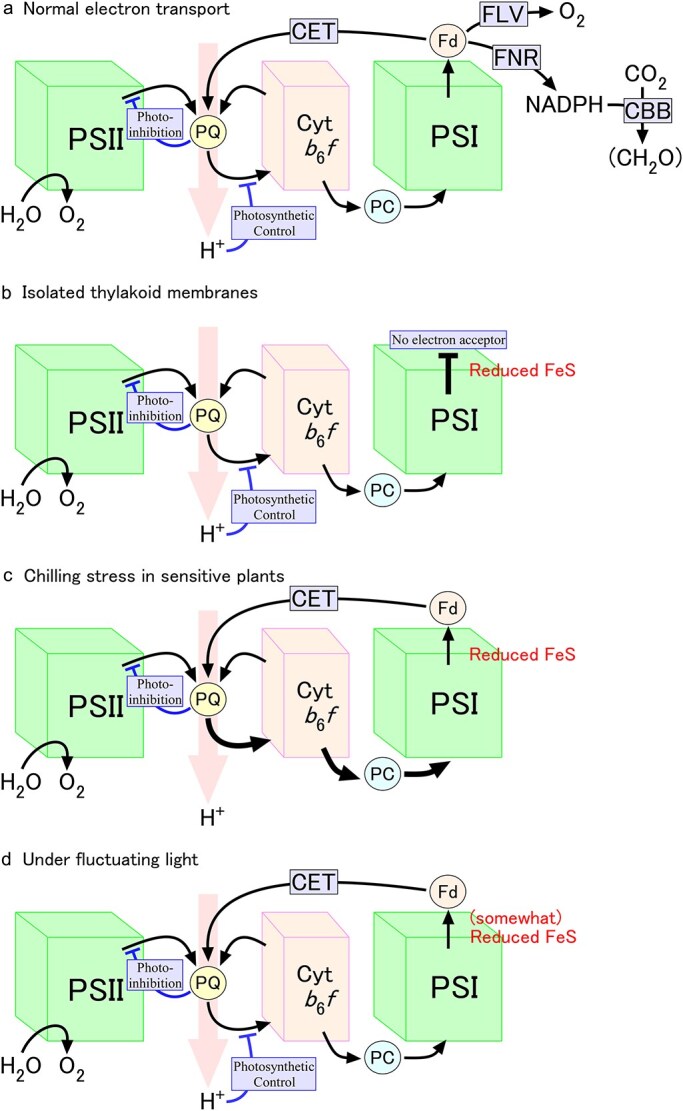
The lack of regulatory mechanisms under stress conditions. Under normal conditions, several different mechanisms work to protect PSI from photoinhibition, e.g. flavodiiron protein (Flv), cyclic electron transport (CET), photosynthetic control, photoinhibition of PSII, and carbon-Benson-Bassham cycle (CBB) itself can be regarded as a kind of protection mechanism (a). Cyclic electron transport and CBB cycle are lost in isolated thylakoid membranes, resulting in the lack of electron acceptors for PSI (b). CBB cycle and photosynthetic control are lost by chilling stress in sensitive plants because of the temperature dependency of CBB cycle and uncoupling of thylakoid membranes, respectively (c). Under fluctuating light (d), the rate of CBB cycle is insufficient to operate as an electron sink, leading to the moderate reduction of PSI electron carriers, which is fatal if cyclic electron transport is not functional.

There are several points that should be considered in the process of ROS production. The actual place of oxygen reduction has been generally assumed to be PSI (Asada 1999). The reduced F_A_ and F_B_, the terminal electron acceptors in the PSI complexes, are relatively stable and have a long half-decay lifetime of around 30 ms attributed to the charge recombination with P700^+^ (Golbeck and Cornelius 1986), so there is plenty of time to react with oxygen. Earlier electron acceptors, including F_X_, have much shorter lifetimes (<1 ms), and withdrawal of electrons by the addition of electron acceptors such as MV is known to require very high concentrations (Sonoike and Terashima 1994). The stable accumulation of the reduced form of earlier electron acceptors of PSI, namely F_X_ and phylloquinone A_1_, usually requires strong reductants in combination with light illumination. In the case of low light-induced photoinhibition of PSI observed in leaves of chilling-sensitive plants under chilling temperatures or in isolated thylakoid membranes, F_A_ and F_B_ are the most probable candidates for the site of oxygen reduction, since earlier electron acceptors are difficult to accumulate under such conditions. During the short but strong light, however, such early electron acceptors can continue to be in a reduced state (Tsuyama and Kobayashi 2009), so there is a chance to react with molecular oxygen. Indeed, it was reported that phylloquinone A_1_ is the principal site of oxygen reduction in PSI complexes isolated from *Chlamydomonas reinhardtii*, at least under high light (a magnitude higher than the growth light) in the presence of a high concentration of plastocyanin, the electron donor to P700 (Kozuleva et al. 2021). This may be a reason for the susceptibility of PSI to fluctuating light or saturating pulses, i.e. short pulses of high light with weak light periods in between (see Fig. 2d).

If we consider the mechanism of PSI photoinhibition based on that in isolated thylakoid membranes, the production site of ˙OH is assumed to be the three FeS since these are the only Fe-containing components of PSI complexes. The initial damaging site should also be these FeS because ˙OH is a highly reactive species that cannot travel long distances. Since the destruction of FeS in chilling-induced PSI photoinhibition was directly shown by the measurements of the electron spin resonance signal of FeS (Sonoike et al. 1995b), the mechanism seems to be common between these two types of photoinhibition induced under relatively low light conditions. The destruction of P700 itself is apparently the secondary effect of initial inhibition in FeS since the chemically determined amount of P700 did not decrease at least in the initial stage of photoinhibition both in chilling-induced photoinhibition (Terashima et al. 1994) and that observed in isolated thylakoid membranes (Sonoike 1995). As for fluctuating light conditions, the photon flux density of the high light phase of the fluctuating light (e.g. around 500 μmol m^−2^ s^−1^) is usually higher than the typical growth light but lower than direct sunlight. The mechanism of the inhibition seems to be more or less similar to the low light-induced PSI photoinhibition since the destruction of FeS prior to the inhibition of P700 was also observed with fluctuating light-induced photoinhibition (e.g. Tiwari et al. 2024). The detailed mechanism of the destruction of PSI, however, has not been reported, at least in parallel with other photoinhibitory conditions.

In the case of photoinhibition by a series of saturating light pulses, the mechanism of the inhibition may be different from that of low light-induced PSI photoinhibition. Shimakawa and coworkers compare the mechanism of photoinhibition between chilling-induced and flash-induced inhibition using the same plant species (cucumber plants) under the same growth conditions and found that FeS is not destroyed by the repetitive saturating pulses, although the destruction of FeS was confirmed in chilling-induced photoinhibition (Shimakawa et al. 2024). The target site of the flash-induced inhibition may be different from that of chilling-induced inhibition, possibly being chlorophyll molecules near the reaction center. It should also be noted that the addition of 2,2,5,7,8-pentamethyl-6-chromanol, a scavenger of singlet oxygen (^1^O_2_), partially protects PSI of isolated intact chloroplasts from photoinhibition by a series of saturating light pulses (Takagi et al. 2016). The involvement of ^1^O_2_ in PSI photoinhibition is controversial, and some reports deny the production of ^1^O_2_ in PSI (Hideg and Vass 1995), but that should depend on the light conditions. Under extreme high light such as 6000 μmol m^−2^ s^−1^, i.e. three-fold of direct sunlight, the early electron acceptors of PSI would be temporarily reduced, leading to the production of ^1^O_2_ that brings about photoinhibition, the mechanism of which is different from photoinhibition induced under the light at more physiological photon flux densities. Thus, under very intense light pulses, both the ˙OH mechanism and the ^1^O_2_ mechanism may be involved in the inhibition (Fig. 1b). Furthermore, in some shade plants such as *Nephrolepis falciformis* fern, the mechanism of photoinhibition seems to be totally different from that discussed above, since the extent of inhibition was larger with simple strong light than with fluctuating light and it increased upon the addition of MV (Huang et al. 2018).

## The Effect of Redox Conditions on PSI Photoinhibition

According to the above-mentioned model, the important factor that influences the rate of PSI photoinhibition is the redox state of the acceptor side of PSI, e.g. the FeS that is the site of ˙OH production. The production rate of ˙OH should be proportional to the product of the two concentrations, [reduced FeS] and [H_2_O_2_]. The addition of MV increases the latter but decreases the former to almost zero, resulting in the suppression of PSI photoinhibition as mentioned above (Sonoike 1996b, Takagi et al. 2016). Thus, the protective role of MV reported in past literature can be at least partly interpreted as the result of FeS oxidation. This indicates that the redox state of FeS is more important than the production rate of H_2_O_2_. Accordingly, the absolute value of electron transport rate is not important for the rate of PSI photoinhibition at least in the case of low light-induced photoinhibition of PSI in chilling-sensitive plants (Terashima et al. 1994) and in isolated thylakoid membranes (Sonoike 1995) where the largest electron sink of Calvin-Benson Bassham (CBB) cycle is lost by decreased temperatures or by the lack of stromal enzymes. It must be noted, however, that the effect of low temperature cannot solely be explained by the temperature dependency of CBB cycle activity, and other factors should be considered (Sonoike 1998, Sonoike 1999). During the high light phase of fluctuating light-induced and pulse light-induced photoinhibition of PSI, the rate of CBB cycle is not high enough to consume reducing power from electron transport chain, resulting in the reduction of PSI electron acceptors as well.

In elucidating the effect of electron flow from PSII on PSI photoinhibition, it is important to consider the influence of the redox state of each electron transport component of PSI on the inhibition. The redox state of the PSI donor side and acceptor side is often estimated by Y(ND) and Y(NA), the parameters so-called “donor side limitation” and “acceptor side limitation,” respectively, determined by the analysis of absorbance change upon P700 reduction/oxidation (Klughammer and Schreiber 1994). Actually, it was generally reported that PSI was photoinhibited under the conditions that P700 is in a reduced state, i.e. Y(ND) is low. Since the reduced P700 is stable in the dark, it in itself does not act inhibitory by any means. Thus, the relationship between Y(ND) and PSI photoinhibition has sometimes been interpreted by the protective role of P700^+^ (for a review, see Miyake 2020) rather than the inhibitory action of the reduced state of P700. P700^+^ was reported to have potential to protect PSI through its activity of quenching excess energy (Nuijs et al. 1986, Trissl 1997). Furthermore, the triplet state of P700 (^3^P700) is relatively stable and not harmful (for a review, see Karapetyan 2008) so that the charge recombination between P700^+^ and electron acceptors should protect PSI rather than inhibit it (Rutherford et al. 2012) through the elimination of potentially dangerous reduced electron acceptors with low reduction potential.

Since an increase in Y(ND) is usually accompanied by a decrease in Y(NA), the protective role of Y(ND) can be also simply explained by the more oxidized electron transport components on the PSI acceptor side. Yamamoto and Shikanai (2019) tried to separate the effects of Y(ND) from that of Y(NA) by comparing the *proton gradient regulation 1* (*pgr1*) mutant showing strong downregulation of donor side electron transport and the flavodiiron protein (Flv)-overexpressing plants showing more oxidized acceptor side of PSI. The separation, however, was not possible since the downregulation of donor side electron transport was also induced by the overexpression of Flv through the Flv-dependent pseudo-cyclic electron transport (Yamamoto et al. 2016). Takagi et al. (2019) compared the high light acclimation of six varieties of wheat and found that there were differences in the sensitivity to PSI photoinhibition among the varieties as well as between high- and low-light acclimated plants. In this report, Y(ND) differs without significant changes in Y(NA), so it looks possible to separate the effects of redox states on the susceptibility to photoinhibition. However, the difference in the sensitivity to photoinhibition could not be explained by the difference in Y(ND) or in Y(NA), and additional factor(s) may be important in this case. Thus, it is rather difficult to conclude the significance of the redox condition of the donor/acceptor side in PSI photoinhibition solely from photosynthetic parameters such as Y(ND) and Y(NA). However, it was reported that the accumulation of plastid terminal oxidase, which withdraws electrons from the plastoquinone (PQ) pool to reduce molecular oxygen, resulted in the oxidation of P700 in the WT background but not in the *pgr5* mutant (Zhou et al. 2022). Apparently, simple manipulation of redox state of donor side of PSI is not enough to change the redox condition of PSI complexes. The result supports the idea that the acceptor side of PSI is more important than the donor side as a factor influencing the extent of PSI photoinhibition (see next section).

## Protective Mechanisms for PSI

### Necessity of protective mechanisms

It seems that PSI is well protected in natural environments (Fig. 2a). Until the first report of selective PSI photoinhibition (Terashima et al. 1994), nearly 40 years had passed since the initial definition of photoinhibition in 1956 (Kok 1956). The conditions that induce selective PSI photoinhibition, namely, chilling-sensitive plants at chilling temperatures, isolated thylakoid membranes without soluble proteins, and mutants of cyclic electron transport under fluctuating light, can all be regarded as conditions that lack some kinds of protective mechanisms (Fig. 2). There are several reasons for the necessity of such strict and multiplex protection. First, the turnover of PSI proteins is so slow that the recovery takes a long time, e.g. for a week (Kudoh and Sonoike 2002a, Zivcak et al. 2015), once the reaction center proteins are degraded during photoinhibition (Sonoike et al. 1997). Thus, photoinhibition of PSI should be more harmful than that of PSII in the long term, even if both photosystems are equally photoinhibited. Furthermore, once PSI is photoinhibited, the function of both linear and cyclic electron transport should be lost, and ATP that is necessary for the cellular function cannot be synthesized. The effect of PSI photoinhibition is not limited to electron transport. The absence of PSI may result in a decrease in the activities of CBB cycle enzymes through the disorder of its redox regulation (Zivcak et al. 2015). As for the acclimatory responses, however, it was reported that both short-term and long-term responses can be induced by the selective photoinhibition of PSI subsequent to the fluctuating light treatment (Lempiäinen et al. 2022) in addition to the initial acclimatory responses induced by the fluctuating light itself (Alter et al. 2012).

PSII photoinhibition, in contrast to PSI photoinhibition, is reversible and can be repaired in a relatively short time. Moreover, PSII photoinhibition itself can be regarded as the protective mechanism of PSI from photoinhibition (Sonoike 1996a). The suppression of electron transport due to PSII photoinhibition should protect PSI from photoinhibition, as a kind of autonomous protecting mechanism, since the inhibition of PSI blocks the electron transport downstream of PSII, which, in turn, induces photoinhibition of PSII (Fig. 2a). Furthermore, PSI photoinhibition blocks the synthesis of ATP, which is necessary for the repair of PSII (Nishiyama and Murata 2014), thus further enhancing the photoinhibition of PSII. In this context, it should be noted that the relative quantum yield of PSI is generally higher than that of PSII under normal conditions in many photosynthetic organisms (see, e.g. Fig. 2 in Terashima et al. 1994). It was reported that a shade plant, *Alocasia odora*, which is tolerant to PSI photoinhibition induced by fluctuating light, shows low PSII/PSI ratio that could be the cause of the tolerance to photoinhibition through relatively smaller reducing pressure from PSII to PSI (Terashima et al. 2021). Transfer of excitation energy from PSII to PSI by so-called “spillover” should also result in smaller reducing pressure from PSII (Terashima et al. 2025). Photosystem stoichiometry and spillover may have potential impact on the extent of PSI photoinhibition, but these mechanisms should be considered to be constitutive at least for land plants.

In the case of cyanobacteria, the PSI/PSII ratio is around 10 on a chlorophyll basis in many species. Although these characteristics can also be regarded as a kind of protective mechanism to reduce the electron pressure toward PSI, it takes 12–24 h to modulate photosystem stoichiometry even in cyanobacteria (Hihara et al. 1998). To avoid possible photoinhibition of PSI, it is beneficial to set the default ratio of PSI/PSII at more than one, and the imbalance can be adjusted either by cyclic electron transport or by state transition. It may be worth mentioning that cyanobacterial mutants defective in the regulation of photosystem stoichiometry cannot survive under continuous high light (Sonoike et al. 2001), although the actual mechanism of the growth inhibition is not yet known. At all events, photoinhibition of PSI is the damage that should be avoided, even at the cost, while that of PSII can be regarded as a regulatory mechanism of photosynthesis. Actually, it was reported that PSI as well as cytochrome *b*_6_*f* complexes are fully functional even after the illumination of tobacco leaves in the presence of lincomycin, i.e. in the absence of protein synthesis, under the condition that PSII is totally photoinhibited (Chow and Hope 1998).

To understand the regulation of photosynthetic electron transport, it is important to know its rate-limiting step. The rate-limiting step of electron transport is the oxidation of the PQ pool, the rate of which is about 20 ms (Stiehl and Witt 1968). The next slowest step is the S_3_ to S_0_ (about 2 ms) in the S-state cycle of oxygen-evolving complex (OEC) in PSII (Klauss et al. 2012). Most of the other steps in electron transport are more than one order of magnitude faster compared with these two steps (but see, e.g. Kirchhoff et al. 2011). This simple fact tells us two things. First, electron carriers in the cytochrome *b*_6_*f* complex, plastocyanin, and P700 should be oxidized under steady-state conditions if sufficient light is applied and the acceptor side of PSI is not limited. In the same condition, the PQ pool should be in a reduced state. Thus, simple high light conditions may induce PSII photoinhibition, but not so much PSI photoinhibition. Secondly, the regulatory step of electron transport should be the cytochrome *b*_6_*f* complex, since regulations at other than the rate-limiting step are useless. Actually, the cytochrome *b*_6_*f* complex has been assumed to be the site of regulation by so-called “photosynthetic control,” as we will see in the next section.

In contrast to the case of PSII, the energy dissipation within the PSI antenna and/or reaction center complex is not an efficient way to avoid photoinhibition, since the reducing equivalent is first produced in PSII, not in PSI (but see, e.g. Tiwari et al. 2016). In this context, the direct protective role of P700^+^ (and the high Y(ND)) through energy dissipation should have a smaller impact on the extent of photoinhibition than the reduction of FeS (and the high Y(NA)). Furthermore, it was reported that the thermal dissipation in PSI estimated by the photoacoustic technique increased when P700 was reduced (Bukhov and Carpentier 2003), the result also questioned the direct protective role of P700^+^. In any event, oxidizing the acceptor side components of PSI is essential for its protection, and for that purpose, the regulation of electron transport is important. The detailed mechanism of such regulation is discussed in the following sections and summarized in Fig. 2.

### Photosynthetic control

One way to oxidize the acceptor side of PSI is to decrease the influx of electrons to PSI. Photosynthetic control is an analogous concept to “respiratory control” that was discovered in the early 1950s (e.g. Lardy and Wellman 1952). Respiratory control was originally defined as the increase in respiratory rate upon the addition of ADP and was later explained by the coupling of ATP synthesis and electron transport mediated by the proton motive force, i.e. the chemiosmotic theory by Peter Mitchell (Mitchell 1961). A similar enhancing effect by the addition of ADP was observed in broken chloroplasts and was termed as photosynthetic control (e.g. Arnon et al. 1958, West and Wiskich 1968, Rumberg and Siggel 1969). At the early stage of the studies of both respiratory and photosynthetic control, the mechanism of the control was ascribed to the coupling of electron transport and ATP synthesis. If the building of proton gradient is obligatorily coupled with the electron transport, it is natural to assume that the rate of quinone (Q) pool oxidation (or reduction) decreases upon an increase (or decrease) of proton concentration at the site of reaction. The rate of redox reaction of quinone, Q + 2e^−^ + 2H^+^ ↔ QH_2_, should certainly depend not only on [Q] and [QH_2_] but also on [H^+^]. Besides this type of “kinetic” control, allosteric type control through the binding of adenine nucleotides to subunit IV of cytochrome *c* oxidase was also proposed for respiratory control (Kadenbach and Arnold 1999). In the case of photosynthetic control, the proton gradient, not ATP/ADP itself, is important for the regulation (Kobayashi et al. 1979). One of the regulatory points was shown to be an iron–sulfur protein (ISP) of the cytochrome *b*_6_*f* complex (Jahns et al. 2002). Single mutation of ISP resulted in the change in pH dependency of the electron transport rate through the change in the pKa of His128 (a ligand to the iron–sulfur cluster) of this protein, resulting in the protection of PSI (Yamamoto and Shikanai 2019). The disulfide bridge in the ISP of cytochrome *b*_6_*f* complex was suggested to act as a regulator of the pKa of His128 in a part of the redox regulation of photosynthetic control (Hald et al. 2008). At any rate, the absence of cyclic electron transport has been repeatedly reported to result in the photoinhibition of PSI especially under fluctuating conditions, so the protective role of proton gradient for PSI is doubtless. In this context, it is worth mentioning that ATPase was reported to be uncoupled after chilling treatment of chilling-sensitive plants under illumination (Terashima et al. 1991a, 1991b). Under such conditions, ΔpH formation and photosynthetic control should be lost, and this may partly explain the sensitivity of PSI to photoinhibition in chilling-sensitive species (Fig. 2c).

There may be another kind of control independent of the proton gradient. Using the cyanobacterial mutant of Flv1/Flv3 (see the next section), it was shown that PQ pool reduction induced by the deprivation of inorganic carbon was positively correlated with the oxidation state of P700 (Shaku et al. 2016). The addition of inorganic carbon, which should result in the consumption of ATP and NADPH in the CBB cycle, induces the reduction of P700, i.e. the increase of electron flow. On the other hand, P700 remained in its oxidized form when electrons were withdrawn through a peroxidase reaction, which does not consume ATP, and thus the proton gradient was maintained (Shimakawa and Miyake 2018). The results can be partly interpreted by a kind of photosynthetic control, possibly at the PQ reduction site of cytochrome *b*_6_*f* complexes. Apparently, the mode of photosynthetic control is not limited to the simple regulation by proton gradient, and its mechanism is quite complex, probably involving several regulatory steps, including redox regulation of the acceptor side of PSI (for a review, see Degen and Johnson 2024). The entire mechanism of photosynthetic control is out of the scope of this review, but the downregulation of the electron transport from PSII through the cytochrome *b*_6_*f* complexes should be the simplest protective mechanism of PSI from photoinhibition.

### Electron sinks

Another way to oxidize the acceptor side of PSI is to increase the outflux of electrons from PSI. In intact photosynthetic systems, the largest sink of electrons is the CBB cycle, and the susceptibility of isolated thylakoid membranes, which lack stromal enzymes, to PSI photoinhibition can be partly explained by this fact (Fig. 2b). If there are some alternative electron sinks other than the CBB cycle, they should also work as protective mechanisms of PSI from photoinhibition, especially under decreased carbon fixation at low temperatures or under low CO_2_ concentration. This fact also partly explains the mechanism of chilling-induced photoinhibition of PSI (Fig. 2c). Some of the electron transport components, PQ or Fd/NADP^+^, are called “pools” since their stoichiometry is higher compared with other components in the transmembrane complexes and can act as buffers in electron transport. The number of component molecules in these pools is, however, not so large as to function as an electron sink, and a short saturating pulse light of subsecond order is enough to reduce all the components of electron transport. The only molecule that can act as an electron sink for photosynthesis other than carbon dioxide is molecular oxygen. Thus, oxygen reduction is a protective mechanism for PSI provided that the damage from ROS can be avoided, as extensively argued in past literature (e.g. Asada 1999). The so-called “water–water cycle” is a pathway that eliminates reducing power by reducing molecular oxygen to water. Water–water cycle does not seem to play an important role in many plant species but oxygen-dependent P700 oxidation was observed in *Cameria* and the presence of water–water cycle was assumed to protect PSI from photoinhibition in that case (Huang et al. 2019). Photorespiration can be also regarded as a kind of electron sink that protects PSI (e.g. Wada et al. 2018).

In the case of cyanobacteria, photosynthetic prokaryotes, photosynthetic and respiratory electron transport chains share some components in the same thylakoid membranes. Therefore, the excess electrons can be transferred to molecular oxygen through the respiratory cytochrome *c* oxidase. This may also work as a simple protective mechanism. Another route of electron transport to molecular oxygen is Flv. In cyanobacteria, Flv1 and Flv3 proteins accept electrons from the reducing side of PSI and reduce oxygen without producing ROS (Helman et al. 2003). In the absence of Flv1/Flv3, P700 was in its reduced form after dark acclimation or inhibiting CBB cycle (Helman et al. 2003), so the electron flow to these Flvs has a significant role as an electron sink under stress conditions, such as inorganic carbon deprivation (Allahverdiyeva et al. 2011). Furthermore, it was demonstrated that Flv1 and Flv3 are essential for growth under fluctuating light conditions and for avoiding photoinhibition of PSI (Allahverdiyeva et al. 2013). The homologs of Flv proteins are present in green algae, mosses, and lycophytes (Zhang et al. 2009) and gymnosperm but not seen in angiosperm. The rate of oxygen reduction was actually shown to be lower in angiosperm than in gymnosperm (Shirao et al. 2013). The protective role of Flv should have been replaced by some other mechanisms during evolution (for a review, see Allahverdiyeva et al. 2015). In *Arabidopsis*, oxygen reduction facilitated by an isoform of the PsaE subunit of PSI was proposed to have a protective role against PSII photoinhibition (Krieger-Liszkay et al. 2020), but its involvement in the protection of PSI from photoinhibition is not known.

### Cyclic electron transport

Cyclic electron transport is the electron flow from the PSI acceptor side to the PSI donor side through the PQ pool and cytochrome *b*_6_*f* complexes. Although the number of total electrons around PSI does not change by the cyclic electron flow, it should protect PSI in several ways. First, cyclic electron transport oxidizes the PSI acceptor side in compensation for the reduction in the PQ pool, since the rate-limiting step of photosynthetic electron transport is PQ oxidation at cytochrome *b*_6_*f* complexes, as mentioned above. Oxidation of the PSI acceptor side should alleviate photoinhibition of PSI. The reduction of the PQ pool may aggravate the photoinhibition of PSII, but this in itself may protect PSI photoinhibition as described above. Secondly, the formation of a proton gradient or reduction of the PQ pool by the action of cyclic electron transport should induce the downregulation of photosynthetic electron transport as described above. Thirdly, the cyclic electron flow can induce non-photochemical quenching in PSII through extra ΔpH formation, which should result in reduced electron flow from PSII. It was reported, however, that the induction of non-photochemical quenching may not be so important, since the *npq4* mutant lacking PsbS protein showed strong downregulation of electron transport in the absence of non-photochemical quenching in PSII (Tikkanen et al. 2015). Since soluble ferredoxin is lost in isolated thylakoid membranes, there should be no cyclic electron transport. This may also contribute to the sensitivity of PSI in isolated thylakoid membranes to photoinhibition (Fig. 2b).

If cyclic electron transport is protective for PSI, the mutants of cyclic electron transport components such as PGR5 should be susceptible to PSI photoinhibition. Actually, the *Arabidopsis chlororespiratory reduction 2* (*crr2*) mutant defective in cyclic electron flow through the NADH dehydrogenase-like (NDH) complexes under repetitive saturating pulse (Tsuyama and Kobayashi 2009) as well as the *pgr5* mutant defective in Antimycin A sensitive cyclic electron transport under fluctuating light conditions (Tikkanen et al. 2010) showed lethal phenotype. Since the phenotypes of the *Arabidopsis ndho* mutant or the *crr2* mutant defective in cyclic electron flow through the NDH complexes are not so much different from that of WT plants under fluctuating light conditions (Suorsa et al. 2012, Kono et al. 2014), the importance of NDH complexes in protecting PSI seems to be smaller compared to the Antimycin A sensitive cyclic electron transport. In the case of rice plants, a decrease in PSI and dry weight was observed in both the *pgr5* mutant and the *crr6* mutant (Yamori et al. 2016). The inhibition of PSI upon the addition of antimycin A was also reported in tobacco leaves (Chow and Hope 1998), confirming the importance of cyclic electron transport in protecting PSI.

The involvement of PGR5 in cyanobacteria is rather controversial. While a similar role as in plant cyclic electron transport was reported in some cases of cyanobacteria (Yeremenko et al. 2005, Dann and Leister 2019), no significant phenotypes were found in other reports (Allahverdiyeva et al. 2013, Sétif et al. 2020). In any event, cyanobacterial cyclic electron transport is essential for the active carbon concentration mechanism that is necessary for photosynthesis under low CO_2_ conditions (for a review, see Fukuzawa et al. 2012) as well as for active ion efflux that is necessary for the growth under salinity conditions (e.g. Hibino et al. 1996).

### State transitions

State transition is one of the well-known regulations of photosynthetic antenna systems, found in 1969 (Bonaventura and Myers 1969, Murata 1969). In the regulation of land plants and green algae, the reduction of the PQ pool activates the kinase of light-harvesting complex II (LHCII), resulting in the reallocation of energy absorbed by LHCII to PSI and PSII. A similar regulatory mechanism of energy allocation between the two photosystems is also observed in red algae and cyanobacteria, having a phycobilisome antenna system. The state transition of phycobilisome-containing organisms is also triggered by the reduction of the PQ pool, despite the difference in their antenna systems. The mechanism of cyanobacterial state transition involves specific components (e.g. Fukunaga et al. 2024) and is less understood compared with the case of land plants. State transition was originally considered as a mechanism to balance the activities of PSI and PSII for optimizing the efficiency of photosynthesis under low light conditions. However, the mechanism can also be effective for the regulation of electron transport under high light conditions, both in *Arabidopsis* (Bellafiore et al. 2005) and cyanobacteria (Fujimori et al. 2005).

Under fluctuating conditions, the *Arabidopsis* mutant *stn7* lacking state transition showed a slow growth phenotype (Tikkanen et al. 2010). This phenotype, together with the decrease in PSI, can be attributed to the result of excess reduction of the electron transport chain, which should be caused by the lack of state transition, since the phenotype is also observed in the *psaL* mutant, another mutant defective in state transition (Grieco et al. 2012).

### Growth light conditions

Although some extent of photoinhibition of PSI has been observed in WT plants, significant phenotypes were only observed in the mutants defective in protective mechanisms (Tikkanen et al. 2010, Suorsa et al. 2012, Kono et al. 2014, Yamori et al. 2016). As the cause of the robustness of PSI in natural environments, the presence of far-red light in the sunlight was proposed (Kono et al. 2017). Since the far-red light acts as the light that preferentially excites PSI, it should oxidize the electron transport chain between PSI and PSII, which alleviates PSII photoinhibition. Simple movement of electrons from the donor side to the acceptor side of PSI, however, should aggravate rather than alleviate PSI photoinhibition as discussed above. Because the effect of far-red light was lost in the absence of the activity of NDH complexes (Kono et al. 2017), not the far-red light itself but the cyclic electron transport induced by it seems to be essential for its protective role. The high growth light itself was also protective for PSI (Sonoike et al. 1995a, Kono et al. 2017), although the mechanism is not clear.

### The choice of tactics

Apparently, the above-mentioned tactics to protect PSI from photoinhibition are used differently in different organisms. Although the Flv mechanism used in cyanobacteria and other photosynthetic organisms seems to be very efficient for the protection of PSI, it is absent in angiosperms, and cyclic electron transport is used in its place. Because cyclic electron transport is also active in cyanobacteria or eukaryotic algae, the cause of the difference should be ascribed to the difference in electron sink. If you look at the total metabolic flow of photosynthetic organisms, the flow to reduce carbon dioxide, i.e. the CBB cycle, dominates over other flows in land plants because of the accumulation of large amounts of cellulose. Thus, additional electron sinks in land plants may have only a minor effect in the regulation of photosynthesis in the presence of the CBB cycle as a huge electron sink, leading to the preference for cyclic electron transport for protection tactics. In the case of microorganisms, however, the electron sink such as Flv mechanism could be a significant regulator of electron flow. The difference between angiosperm and gymnosperm is more difficult to explain, but the limitation of photosynthesis due to tracheid tissue in gymnosperm may lead to the relative importance of electron sink as a regulatory tactic.

## Photoinhibition and Redox Potentials of Electron Carriers

At the end of this review, let us consider the relationship between photoinhibition and the reduction potential of electron carriers. Oxygenic photosynthetic electron transport is a chain of redox reactions: the reduction potential of PSII is high (positive) enough to oxidize water to oxygen, while that of PSI is low (negative) enough to reduce NADPH, which is used to reduce carbon dioxide in the CBB cycle. The different reduction potentials of these two photosystems make it possible to use water as an electron donor on one hand and to make reducing power on the other.

It seems that different reduction potentials lead to different mechanisms of photoinhibition in the two photosystems. PSII is the only biological system that has the ability to oxidize water to molecular oxygen, and its electron transport components on the donor side are strong oxidants with a standard reduction potential larger than +0.8 V, which can oxidatively destroy neighborhood biological components. In the “standard” models of PSII photoinhibition, charge recombination between oxidized reaction center chlorophyll of PSII (P680^+^) and reduced electron acceptors produces triplet state of P680 (^3^P680), leading to the subsequent production of singlet oxygen (^1^O_2_), which is the damaging species to PSII. By contrast, the acceptor side components of PSII have moderate standard reduction potential; around −0.6 to 0 V. Since the standard reduction potential of oxygen reduction reaction is −0.33 V, oxygen can be theoretically reduced by PSII, but it is generally supposed that oxygen reduction at PSII is not a major source of ROS (Asada 1999). Thus, in PSII, the photosystem with high reduction potential, oxidation of the donor side components is more dangerous than the reduction of acceptor side components.

The situation is totally different in PSI. Electron acceptors of PSI have negative standard reduction potential around −0.5 to −1 V, which enables them to reduce oxygen to produce O_2_˙^−^, which is generally assumed to be a major site of ROS production (Asada 1999). On the other hand, P700, the reaction center chlorophyll of PSI, has a moderate standard reduction potential at +0.4 V; thus, the oxidized P700 (P700^+^) is not supposed to be dangerous as discussed above. As a result, electron donation to PSII acts protectively while that to PSI acts inhibitory to photosynthesis. Since electron donation to the PSII reaction center eliminates the dangerous P680^+^, the inability of electron donation to the PSII reaction center through, e.g. the destruction of the Mn cluster of OEC by UV irradiation, should bring about photoinhibition, which is a part of the mechanism of the two-step model of PSII photoinhibition (Tyystjärvi 2013, Kono et al. 2024). On the other hand, electron donation to PSI not only eliminates protective P700^+^ but also leads to the reduction of dangerous acceptor side components (Sonoike 1996a), which is a general scheme of PSI photoinhibition, discussed above.

## Future Perspectives

We now have a framework for discussing the mechanisms of photoinhibition of PSI induced under different conditions, as described above. A more detailed mechanism concerning ROS species involved in the inhibition or the precise contribution of Y(ND) and Y(NA) to the sensitivity to photoinhibition should be further pursued and understood within the current framework. The role of cyclic electron transport in the protection of PSI should also be more precisely examined with the protective effect of far-red light. At the same time, additional issues should also be pursued that differ from the viewpoints mentioned above.

One such viewpoint is the ecological and physiological consequence of the PSI photoinhibition (for a review, see Kono et al. 2024). Once induced, PSI photoinhibition affects photosynthesis in the long term, since the recovery from PSI photoinhibition is very slow (Kudoh and Sonoike 2002a). Photoinhibition of PSI caused subsequent acclimatory responses due to the decreased activity of PSI (Lempiäinen et al. 2022). Thus, it is necessary to pay attention to the extent of PSI photoinhibition when physiological aspects of photosynthesis under stress conditions are discussed. When dealing with the consequence of the PSI photoinhibition, the species difference should also be considered, since the susceptibility of PSI to photoinhibition is totally different between chilling-sensitive and chilling-tolerant species (Sonoike 1996a) as well as among different cultivars in one species (Takagi et al. 2019).

The consequences of PSI photoinhibition cannot be limited to the acclimatory responses. The damaged PSI complexes should be removed, since absorption of light energy without active photosynthesis is very dangerous. Actually, the damaged PSI reaction center proteins are degraded after photoinhibition together with bound chlorophyll molecules (Kudoh and Sonoike 2002a). Since the active PSI complexes are not degraded, there must be some mechanism that distinguishes active and inactive PSI, and degradation takes place only in the latter. The nature of this selective degradation mechanism should also be the subject of future research. Some other physiological responses, such as those induced by retrograde signaling with the involvement of ROS species produced during the inhibition of PSI, would be possible, but we don’t have any evidence for such phenomena.

Finally, the nature of the chilling sensitivity, which was the starting point of the study on PSI photoinhibition in 1994, remains unsolved. The presence of critical temperature, i.e. the temperature below which the inhibition is induced, suggests the involvement of physical transition such as phase separation of lipid membranes, since the typical temperature dependence of chemical reaction cannot explain the sharp temperature dependence of photoinhibition (Sonoike 1998). This long-standing but critical question should also be pursued in the future.

## Conclusion

Photoinhibition of PSI can be induced under several different conditions. The fundamental requirements for PSI photoinhibition are common among different types of photoinhibitions, but the mechanisms of the inhibition seem to differ, at least between low light-induced inhibition observed in chilling-sensitive plants or isolated thylakoid membranes, and high light-induced inhibition observed in repetitive light pulse treatment. In the former, the reduction of FeS and production of ˙OH is a key factor in the inhibition, while the reduction of earlier electron acceptors of PSI and production of ^1^O_2_ seems to play an important role in the latter. In all types of PSI photoinhibition, however, reduction of PSI acceptor side components is a critical requirement, and downregulation of electron flow from PSII, including photoinhibition of PSII, is important to protect PSI. Different characteristics of photoinhibition of PSI and PSII can be ascribed to the different reduction potentials of the two photosystems. Not only the precise mechanism of PSI photoinhibition but also the physiological and ecological consequences of the inhibition must be pursued in the future.

## Data Availability

No new data were generated or analyzed in support of this research.
